# Artificial neural network identification of exercise expiratory flow-limitation in adults

**DOI:** 10.1038/s41598-023-44331-z

**Published:** 2023-10-11

**Authors:** Hans Christian Haverkamp, Peter Luu, Thomas W. DeCato, Gregory Petrics

**Affiliations:** 1 Department of Nutrition and Exercise Physiology, Washington State University-Spokane Health Sciences, Elson S. Floyd College of Medicine, 412 E. Spokane Falls Blvd., Spokane, WA 99202-2131 USA; 2 Department of Medical Education and Clinical Sciences, Washington State University-Spokane Health Sciences, Elson S. Floyd College of Medicine, Spokane, WA USA; 3 Division of Respiratory & Critical Care Physiology & Medicine, Harbor-UCLA Medical Center and the Lundquist Institute for Biomedical Innovation, Torrance, CA USA; 4 Department of Mathematics, Vermont State University-Johnson, Johnson, VT USA

**Keywords:** Respiration, Machine learning

## Abstract

Identification of ventilatory constraint is a key objective of clinical exercise testing. Expiratory flow-limitation (EFL) is a well-known type of ventilatory constraint. However, EFL is difficult to measure, and commercial metabolic carts do not readily identify or quantify EFL. Deep machine learning might provide a new approach for identifying EFL. The objective of this study was to determine if a convolutional neural network (CNN) could accurately identify EFL during exercise in adults in whom baseline airway function varied from normal to mildly obstructed. 2931 spontaneous exercise flow-volume loops (eFVL) were placed within the baseline maximal expiratory flow-volume curves (MEFV) from 22 adults (15 M, 7 F; age, 32 yrs) in whom lung function varied from normal to mildly obstructed. Each eFVL was coded as EFL or non-EFL, where EFL was defined by eFVLs with expired airflow meeting or exceeding the MEFV curve. A CNN with seven hidden layers and a 2-neuron softmax output layer was used to analyze the eFVLs. Three separate analyses were conducted: (1) all subjects (*n* = 2931 eFVLs, [GR_ALL_]), (2) subjects with normal spirometry (*n* = 1921 eFVLs [GR_NORM_]), (3) subjects with mild airway obstruction (*n* = 1010 eFVLs, [GR_LOW_]). The final output of the CNN was the probability of EFL or non-EFL in each eFVL, which is considered EFL if the probability exceeds 0.5 or 50%. Baseline forced expiratory volume in 1 s/forced vital capacity was 0.77 (94% predicted) in GR_ALL_, 0.83 (100% predicted) in GR_NORM_, and 0.69 (83% predicted) in GR_LOW_. CNN model accuracy was 90.6, 90.5, and 88.0% in GR_ALL_, GR_NORM_ and GR_LOW_, respectively. Negative predictive value (NPV) was higher than positive predictive value (PPV) in GR_NORM_ (93.5 vs. 78.2% for NPV vs. PPV). In GR_LOW_, PPV was slightly higher than NPV (89.5 vs. 84.5% for PPV vs. NPV). A CNN performed very well at identifying eFVLs with EFL during exercise. These findings suggest that deep machine learning could become a viable tool for identifying ventilatory constraint during clinical exercise testing.

## Introduction

Clinical exercise testing is commonly used to identify causes of unexplained or exertional dyspnea and to identify the presence of ventilatory limitations during exercise^1^. Expiratory flow-limitation (EFL) is one contributor to exercise ventilatory constraint that occurs when an increase in pleural pressure is not met with an increase in expiratory flow. In pulmonary disease patients with narrowed airways, EFL can develop at light exercise work rates and with only modest increases in minute ventilation^2–4^. In healthy populations, EFL can occur during exercise at high work rates in well-trained persons^5^. In patients with obstructive lung disease, it is axiomatic that EFL occurs at lower expired airflows than in healthy persons.

In practice, EFL occurs when tidal expiratory airflow meets the maximum possible airflow at any lung volume, as defined by the maximal expiratory flow-volume curve (MEFV). This method for measuring EFL was developed by Hyatt in 1961 and will be referred to as the “Hyatt method”^6^. Exercise EFL is associated with several negative outcomes. EFL can limit exercise ventilation and capacity in both healthy adults and in patients with pulmonary disease^7–10^. Operational lung volumes are increased consequent to EFL, typified by an increased end-expiratory lung volume (EELV)^7,11^. This is disadvantageous, as it increases ventilatory work and worsens dyspnea because of increases in both lung and chest wall elastic work and a shortened diaphragm with reduced force generating capacity^12^.

Identifying EFL is nuanced, time-consuming, and standard metabolic carts do not readily perform the analyses required for the measurement. There are also several limitations inherent in the Hyatt method for measuring EFL. Both thoracic gas compression during the maximal forced expiration^13^ and bronchodilation during exercise^14^ can lead to erroneous measurements of EFL. Accurate placement of the exercise tidal flow-volume loop (eFVL) within the MEFV curve requires precise determination of operational lung volume. Given these limitations, new methods for detecting EFL that do not require comparison of the eFVL with the MEFV curve would be of great benefit.

Deep machine learning might be a viable new approach for identifying EFL during exercise. Deep learning designs artificial neural network (ANN) models. ANNs can be “trained” to predict labels in response to one or more input variables. A convolutional neural network (CNN) is a type of ANN that is particularly adept at detecting shapes and boundaries in time series data. Given the temporal nature of the eFVL and that it essentially consists of a closed, two-dimensional shape with a clear boundary, we reasoned that an appropriately constructed CNN would be able to discern differences in overall shape in eFVLs that are flow-limited *vs*. those that are not. The purpose of this study was therefore to develop and implement a CNN to identify EFL in adults exhibiting a range of baseline airway function. Specifically, the CNNs were trained to identify eFVLs that met or exceeded the expiratory limb of a MEFV curve. Some of the results of these studies have been previously reported in the form of an abstract^15^.

## Methods

### Subjects

The data in this manuscript were collected as part of previous and current research conducted between July, 2010 and February, 2023^2,16–18^. Notwithstanding one abstract with a small number of subjects^15^, the CNN data have not been published previously. All subjects were non-smokers between the ages of 18–50 years. Subjects were fully informed of the procedures, risks, and benefits of the study, and signed an informed consent document. All studies were approved by the Institutional Review Board for research involving human subjects at Northern Vermont University-Johnson and Washington State University-Spokane Health Sciences. Studies were conducted in compliance with the Declaration of Helsinki, except for registration in a database.

All participants had a negative history for chronic illness (excepting asthma), and an absence of respiratory infection during the four-weeks prior to participation. The subjects in this report include healthy adults with normal spirometry and adults with mild airway obstruction. Although most, but not all, of the subjects with mild airflow limitation had a previous asthma diagnosis, they were not categorized as asthmatic in this manuscript. Subjects using oral or inhaled corticosteroids were excluded from participation. Collectively, the subjects were recreationally active, participating in regular aerobic and/or resistance exercise several days each week. All volunteers were instructed to refrain from using short-acting β_2_-agonist for at least eight hours prior to the study and from ingesting products containing caffeine for six hours prior to study. Exercise was avoided for eight hours prior to the lab visit.

### Spirometry

Spirometry was completed in the seated, upright position according to American Thoracic Society and European Respiratory Society standards at the time of testing^19,20^. During each measurement, subjects performed forced vital capacity maneuvers for determination of peak expiratory flow, forced vital capacity (FVC), forced expiratory volume in 1 s (FEV_1_), and forced expiratory flow between 25 and 75% of FVC (FEF_25-75%_). Reference equations are from the Global Lung Initiative^21^. Acknowledging the recent changing perspective on population norms for spirometry^22^, the Caucasian equations were used to determine predicted values for all subjects, regardless of race. Twenty out of the 22 subjects identified as White, one identified as Hispanic, and one identified as Asian.

### Incremental exercise test

Exercise was performed on a magnetically-braked cycle ergometer. Subjects breathed through a two-way, non-rebreathing valve (Hans-Rudolph) with nose clips in place. Separate pneumotachographs (Hans-Rudolph) were used to measure inspiratory and expiratory airflow. A 16-channel analog-to-digital data acquisition system (ADinstruments) interfaced with a laptop computer was used to collect the data. Inspired and expired airflow were continuously collected for generation of eFVLs and inspiratory capacity volumes (IC). Initial workrate was set at 35 watts in women and 50 watts in men. In 19 subjects, workrate was increased by 35 watts every two minutes until the limit of tolerance. In three subjects, workrate was increased by 15 watts each minute until the limit of tolerance.

### Determination of expiratory flow-limitation

The presence of EFL was determined using well-characterized methods that continue to be the standard approach for identifying EFL during exercise^6,23^. Prior to exercise and while seated on the cycle ergometer, subjects performed at least three maximal forced expirations from total lung capacity (i.e. FVC maneuver). A single IC maneuver was performed at the end of each exercise workload. A variable number of breaths preceding each IC were selected for analysis and sampled at 100 Hz. Breaths were selected up to, but never exceeding, 60 s prior to the IC. Breaths with unusual shapes due to cough, sighs, and other anomalies were discarded. The number of eFVLs analyzed among subjects was variable. This variability was due to differences among subjects in the number of workloads completed, breathing frequency, and the incidence of irregular breaths.

The expiratory portion of all eFVLs were placed within the largest pre-exercise MEFV curve after subtracting the IC volume measured at the end of each workload. Exercise flow-volume curves with any portion that met or exceeded the pre-exercise MEFV curve were classified as expiratory flow-limited. Thus, eFVLs that met this criterion were labelled as “EFL” whereas eFVLs that did not meet or exceed the MEFV curve were labelled “non-EFL.” The extent of EFL was quantified as the percentage of tidal volume (VT) meeting or exceeding the MEFV curve (% EFL).

### Artificial neural network

**Figure 1:**
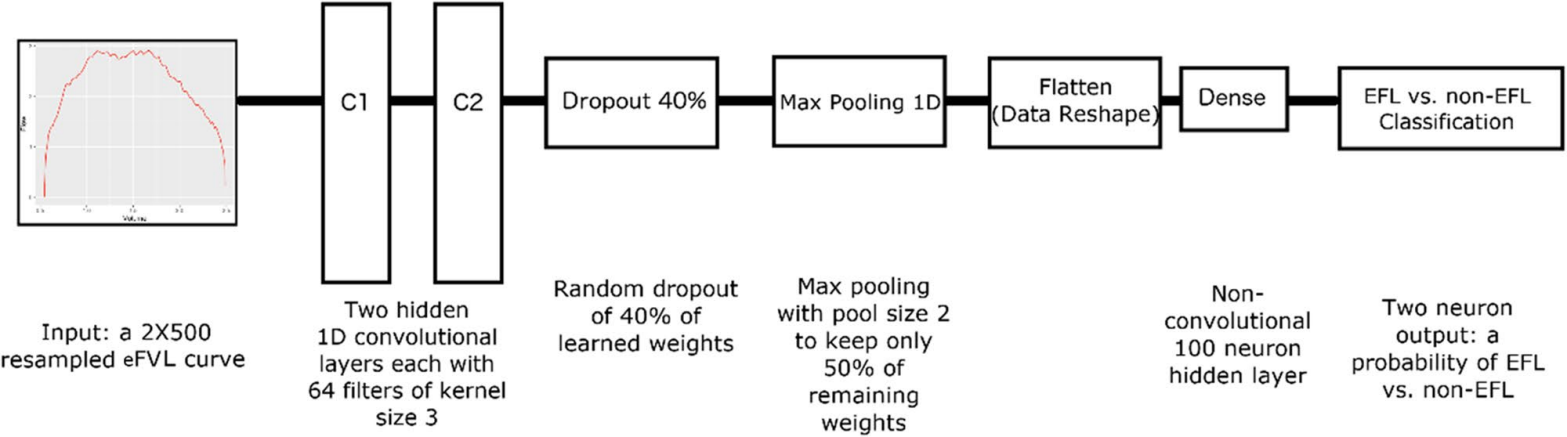
Convolutional neural network architecture. The resampled 2X500 eFVL series is passed through two hidden convolutional layers, each with 64 kernels of size 3. The convolved output, which represents features from the eFVL series, is passed to a random dropout layer, which drops 40% of the learned weights at random to prevent overfitting. The reduced set of weights are then passed to a MaxPooling1D layer of pool size 2 to select the highest weighted learned feature from every other kernel. This is then flattened and passed to a simple neural network of 100 neurons. The final output is the probability of EFL or non-EFL, which is considered EFL if the probability exceeds 0.5 or 50%. *eFVL* exercise tidal flow-volume loop, *EFL* expiratory flow limitation.

The CNN deep-learning based model was developed using Python version 3.9.7 combined with deep learning framework TensorFlow version 2.6.0. Figure 1 depicts the architecture of the CNN. The model is a Sequential TensorFlow model with seven layers. Layers one and two of the model are both Conv1D convolutional layers with 64 filters of kernel size 3 and rectified linear unit (“relu”) activations. Layer three is a dropout layer set to 0.4 thereby randomly setting each input to 0 with 40% probability to encourage each node to be independently useful with the goal of reducing overfitting. Layer four is a MaxPool1D layer with a pool size set to 2 thereby only retaining the most prominent features. When combined with the previous dropout layer, this further decreases the risk of overfitting. Layer five is a flattening layer. Layer six is a traditional Dense neuron layer with 100 neurons. Layer seven is the output layer, which is a Dense layer of 2 neurons, one for each class “EFL” and “non-EFL”. The architecture was adapted from a model used to diagnose problems in internal combustion engines from tailpipe emissions time series^24^.

The order of 2931 spirometry records were randomized by a random permutation, and then a random sampling method was used to distribute the records into training and test data sets with a ratio of 2:1. The training data was further segmented into training and validation sets with a ratio of 9:1.

The original eFVLs were stored as two-channel series of flow and volume in Microsoft Excel. Before deep learning training, all eFVL records were resampled to a standardized 2X500 shape using Forsythe, Malcolm, and Molder (“fmm”) splines. The resampling was performed in R with the spline() method from the stats package.

The input shape of the data to the model was therefore 2X500. The training batch size was set to 250. The number of training epochs was set to 500. The optimizer was set to adam. The loss was set to sparse_categorical_crossentropy. The metric was set to sparse_categorical_accuracy. The learning rate was set to reduce on plateaus of 90 epochs by a factor of 0.5 to prevent overfitting, but the learning rate was not permitted to go below 0.0001. Other settings were set to default. After the training process, the optimal model was selected according to the best sparse_categorical_accuracy.

### Analytic approach

A student’s *t* test was used to compare demographic and pulmonary function variables between two subgroups (normal or low spirometry group; see RESULTS). The eFVL results shown in Table 3 were not normally distributed; a Mann Whitney test was used to compare the variables between the two subgroups. Statistical significance was set at α < 0.05. Success of the final CNN algorithm was determined by analyzing five variables that were calculated from the final confusion matrix output: (1) accuracy; (2) true positive rate (sensitivity); (3) true negative rate (specificity); (4) positive predictive value (precision); and (5) negative predictive value^25^.

## Results

### Participant characteristics

Results for descriptive characteristics and pulmonary function are shown in Table 1. Participants were placed into one of two groups according to their FEV_1_/FVC (GR_NORM_, FEV_1_/FVC > 0.75; GR_LOW_, FEV_1_/FVC < 0.75). There were no differences in anthropometric characteristics between GR_NORM_ and GR_LOW_. Baseline airway function ranged from mildly obstructed in GR_LOW_ to above normal values in GR_NORM_. Percent predicted FEV_1_, FEV_1_/FVC, and FEF_25-75%_ were 22, 20, and 82% higher in GR_NORM_ than GR_LOW_ (*P* < 0.004 for all comparisons).

**Table 1:** Descriptive characteristics and pulmonary function in all subjects, GR_NORM_ and GR_Low_.

Variable	All subjects (*n* = 22)	GR_NORM_ (*n* = 13)	GR_LOW_ (*n* = 9)
Sex	15m/7f.	8m/5f.	7m/2f.
Age, yrs	31.6 ± 9.3	31.4 ± 9.4	31.9 ± 9.6
Height, m	1.73 ± 0.10	1.74 ± 0.10	1.72 ± 0.09
Weight, kg	81.2 ± 16.4	77.1 ± 11.9	87.2 ± 20.8
BMI, kg/m^2^	27.0 ± 4.3	25.7 ± 4.0	28.9 ± 4.2
FVC, l	5.20 ± 1.30 (110 ± 16)	5.21 ± 1.28 (109 ± 17)	5.09 ± 1.40 (103 ± 15)
FEV_1_, l	4.00 ± 1.1 (100 ± 19)	4.32 ± 1.06 (109 ± 18)	3.53 ± 0.97 (86 ± 13)*
FEV_1_/FVC	0.77 ± 0.08 (93 ± 10)	0.83 ± 0.04 (100 ± 5)	0.69 ± 0.04^†^ (83 ± 5)^†^
FEF_25-75%_, l·sec^–1^	3.61 ± 1.55 (87 ± 34)	4.42 ± 1.45 (108 ± 28)	2.43 ± 0.73* (57 ± 10)^†^
PEF, l·sec^–1^	9.12 ± 2.41 (100 ± 20)	9.57 ± 2.35 (106 ± 19)	8.47 ± 2.47 (90 ± 19)
V̇O_2peak_, ml·kg^–1^·min^–1^	43.6 ± 9.4 (126 ± 29)	46.2 ± 10.8 (131 ± 30)	40.0 ± 6.3 (120 ± 28)

Percent predicted values in parentheses.

*BMI* body mass index, *FVC* forced vital capacity, *FEV*_*1*_ forced expiratory volume 1.0 s, *FEF*_*25*-75%_ forced expiratory flow between 25 and 75% FVC, *PEF* peak expiratory flow, *V̇O*_2peak_ peak oxygen consumption, GR_NORM,_ FEV_1_/FVC > 0.75; GR_LOW_, FEV_1_/FVC < 0.75.

*P = 0.001–0.004 vs. GR_NORM_, ^†^*P* < 0.0001 vs. GR_NORM_.

### Convolutional neural network modelling

We performed three separate CNN analyses. In the first analysis, data from all 22 subjects were analyzed collectively. In the second and third analyses, subjects in GR_NORM_ and GR_LOW_ were analyzed separately. Table 2 contains values for accuracy, true positive rate (sensitivity), true negative rate (specificity), positive predictive value (precision, [PPV]), and negative predictive value (NPV). The final CNN algorithm performed remarkably well at correctly identifying eFVLs with EFL in all subjects and in both GR_NORM_ and GR_LOW_. Values for accuracy equaled or exceeded 88% in all three groups. Moreover, values for sensitivity, specificity, PPV and NPV were equal to or greater than 75% in all three groups. In the collective analysis and in GR_NORM_, the CNN performed better at identifying true negatives (higher specificity) than identifying true positives (lower sensitivity). In contrast, in GR_LOW_, the CNN was more successful at identifying true positives (higher sensitivity) than true negatives (lower specificity). In GR_NORM_, NPV was 15.3% higher than PPV (93.5 vs. 78.2%). In GR_LOW_, PPV was 5% higher than NPV (89.5 vs. 84.5%).

**Table 2:** Confusion matrix derivations for all subjects and subjects in GR_NORM_ and GR_LOW_.

	All subjects (*n* = 22)	GR_NORM_ (*n* = 13)	GR_LOW_ (*n* = 9)
	True positive = 302	True positive = 97	True positive = 212
	True negative = 575	True negative = 477	True negative = 82
	False positive = 38	False positive = 27	False positive = 25
	False negative = 53	False negative = 33	False negative = 15
	Total number eFVLs = 968	Total number eFVLs = 634	Total number eFVLs = 334
Variable			
Accuracy (*TP* + *TN*)/(*TP* + *TN* + *FP* + *FN*)	90.6%	90.5%	88.0%
True positive rate (sensitivity) *TP/*(*TP* + *FN*)	85.1%	74.6%	93.4%
True negative rate (specificity) *TN/*(*TN* + *FP*)	93.8%	94.6%	76.6%
Positive predictive value (precision) *TP/*(*TP* + *FP*)	88.8%	78.2%	89.5%
Negative predictive value *TN/*(*TN* + *FN*)	91.6%	93.5%	84.5%

GR_NORM,_ FEV_1_/FVC > 0.75; GR_LOW_, FEV_1_/FVC < 0.75, *eFVL* spontaneous tidal exercise flow-volume loop, *TP* true positive, *TN* true negative, *FP* false positive, *FN* false negative.

### Expiratory flow-limitation

**Figure 2:**
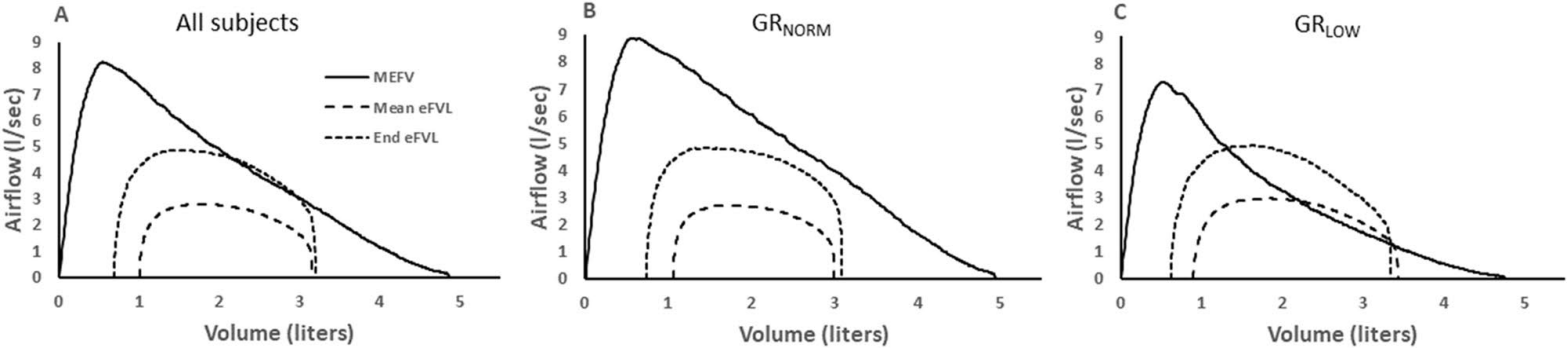
Baseline ensemble-averaged MEFV curves with ensemble-averaged mean and end exercise tidal flow-volume curves (mean eFVL and End eFVL) in (**A**) all subjects, (**B**) subjects with FEV_1_/FVC > 0.75 (GR_NORM_), and (**C**) subjects with FEV_1_/FVC < 0.75 (GR_LOW_). A smaller MEFV curve in GR_LOW_ resulted in substantial expiratory flow-limitation whereas GR_NORM_ subjects, on average, did not develop significant expiratory flow-limitation. *MEFV* maximal expiratory flow-volume curve, *FEV*_*1*_/*FVC* ratio between forced expiratory volume in 1 s and forced vital capacity.

Table 3 lists individual subject values for the total number of eFVLs analyzed, number of eFVLs with EFL, mean exercise % EFL, and end-exercise % EFL. There were more eFVLs with EFL in GR_LOW_ than GR_NORM_; 71.8% (IQR = 0.58) of the eFVLs were EFL in GR_LOW_ whereas 18.8% (IQR = 0.32) of the eFVLs were EFL in GR_NORM_. Additionally, both mean and end-exercise % EFL were higher in GR_LOW_ than GR_NORM_ (*P* < 0.0003 for both comparisons). In Fig. 2A–C, ensemble-averaged mean exercise and end-exercise eFVLs are plotted within the ensemble-averaged MEFV curves in all subjects, GR_NORM_ and GR_LOW_. Note the smaller MEFV curve in GR_LOW_ *vs.* GR_NORM_. In GR_LOW_ subjects, the smaller boundary provided by the MEFV curve resulted in extensive EFL during the exercise, despite similar exercise tidal volumes and expired airflows in GR_LOW_ and GR_NORM_.

**Table 3:** Individual subject values and group mean results for exercise flow-volume loop data in GR_Low_ and GR_NORM_.

Subject no	Total number eFVLs analyzed	Number EFL	Mean exercise, % EFL ± SD	End exercise, % EFL ± SD
Subjects GR_NORM_				
1	92	32	42.3 ± 11.7	40.8 ± 10.9
2	86	23	29.1 ± 13.9	31.1 ± 10.1
3	136	0	na	na
4	102	0	na	na
5	142	140	54.4 ± 21.1	78.0 ± 5.6
6	526	173	17.3 ± 10.0	32.6 ± 9.6
7	269	2	na	7.6 ± 1.7
8	69	1	na	19.2
9	86	11	na	47.8 ± 8.6
10	174	1	na	9.3
11	218	0	na	na
12	88	34	19.8 ± 10.2	25.9 ± 9.4
13	48	0	na	na
Median (IQR)	102.0 (110)	2.0 (33.0)	1.5 (7.0)	9.3 (36.3)
Subjects GR_LOW_				
14	28	28	83.1 ± 13.4	91.5 ± 7.7
15	214	173	86.2 ± 13.0	92.6 ± 0.9
16	40	15	69.3 ± 7.7	73.8 ± 4.4
17	98	92	54.6 ± 16.5	72.6 ± 5.6
18	112	77	50.1 ± 18.2	67.3 ± 4.7
19	117	31	28.7 ± 18.6	41.0 ± 12.3
20	96	95	54.9 ± 15.7	67.6 ± 6.1
21	138	134	52.3 ± 22.2	80.9 ± 7.0
22	173	73	27.7 ± 17.8	53.2 ± 6.7
Median (IQR)	112.0 (87.5)	77.0 (85)*	46.7 (44.9)^†^	68.3 (30.6)^ψ^
All subjects				
Median (IQR)	107.0 (87.3)	29.5 (91.8)	7.6 (46.8)	38.3 (68.6)

*eFVL* exercise tidal flow-volume loop, *EFL* expiratory flow-limitation, *IQR* interquartile range.

GR_NORM,_ FEV_1_/FVC > 0.75; GR_LOW_, FEV_1_/FVC < 0.75.

na indicates that a subject either did not have any eFVLs with EFL (subjects #3, #4, #11, #13), or that all eFVLs with EFL occurred at end exercise (#7, #8, #9, #10).

*P = 0.016 vs. GR_NORM_, ^†^*P* = 0.0002 vs. GR_NORM_, ^ψ^*P* = 0.0003 vs. GR_NORM_.

## Discussion

### Summary

We sought to develop a convolutional neural network (CNN) that accurately identifies exercise tidal flow-volume loops (eFVL) with expired airflow that meets or exceeds the pre-exercise maximum expiratory flow (i.e. EFL). Using 2931 eFVLs from 22 adults in whom airway function varied from normal to mildly obstructed, our CNN performed very well at discriminating between eFVLs that were, and were not, expiratory flow-limited. Our final model achieved an overall accuracy of 90.6%. Subjects were also placed into one of two groups based on their baseline FEV_1_/FVC (GR_NORM_, FEV_1_/FVC > 0.75; GR_LOW_, FEV_1_/FVC < 0.75). The CNN performed equally well in both subgroups, achieving an accuracy, respectively, of 90.5 and 88% in GR_NORM_ and GR_LOW_. Our findings provide proof-of-principle evidence supporting the potential for deep machine learning to identify ventilatory constraints during exercise in adults exhibiting a range of airway function. These encouraging findings thus bode well for development of deep learning methods that can automate identification of exercise ventilatory constraints in clinical exercise testing.

**Figure 3:**
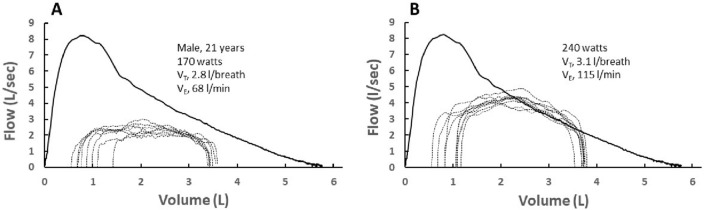
(**A**) individual exercise tidal flow-volume loops (eFVL) plotted within the pre-exercise maximal expiratory flow-volume curve (MEFV) in one subject at a moderate exercise workload. (**B**) individual eFVLs plotted within the pre-exercise MEFV curve in the same subject at a heavy exercise workload. The eFVLs were not expiratory flow-limited at the moderate workload whereas they were flow-limited at the heavy workload. Note that, overall, the second half of the eFVLs that are expiratory flow-limited tend to parallel the MEFV curve. *V*_T_ tidal volume, *V*_E_ minute ventilation.

Our major finding is that a CNN was able to identify if eFVLs were expiratory flow-limited or not with an overall accuracy of 90.6%. Fundamentally, artificial neural networks are pattern-recognition systems that can learn to discern differences in patterns and shapes of data^26^. The high accuracy of our CNN demonstrates that the shape of the expiratory portion of eFVLs differs based on whether the expiration is flow-limited or not. Figure 3A,B depicts two series of eFVLs plotted within the MEFV curve in one GR_LOW_ participant. Panel A depicts eFVLs that were not flow-limited whereas the eFVLs in panel B did demonstrate EFL. Overall, the eFVLs that achieved EFL contain two attributes that are not readily seen in the non-flow-limited eFVLs: (1) a relative peak in the vicinity of their meeting the MEFV curve, and (2) a descending curve with a slope that parallels the MEFV curve.

In addition to the high accuracy, our CNN demonstrated high sensitivity, specificity and both positive- and negative predictive values (Table 2). Although additional studies with a larger number of subjects and data are needed to further determine the viability of a CNN for identifying EFL, the findings in this study are promising.

Recently, Welch et al. developed an approach for identifying EFL that is also based on the geometry of the eFVL^27^. The authors applied a vector-based analysis that compared the contour of eFVLs with the contour of the MEFV curve in a group of healthy adults and a group with airway obstruction. In both subject groups, the incidence of exercise EFL was similar in the contour-based and Hyatt method. Our CNN is based on the same principle that the shape of an eFVL depends on prevailing airway geometry during the breath. Previous studies have also shown, from a qualitative standpoint, that the contours of eFVLs and the MEFV curve are similar when EFL is present^5,28–30^. The success of our CNN at identifying EFL demonstrates that the dynamic interrelationships among airway and pleural pressures, airway collapsibility and caliber, and lung volume result in distinct expired airflow phenotypes in eFVLs that are, and are not, flow-limited.

In the context of this study, sensitivity and specificity indicate the effectiveness of our CNN at correctly classifying eFVLs according to their label (“EFL” or “non-EFL”); the focus is on the screening test, per se^25^. In contrast, PPV and NPV indicate the probability of having EFL (or not) after the CNN testing results are known; the focus is on the patient, per se. Thus, PPV and NPV relate to the utility of the test in practice^25^. We performed the two subgroup analyses to probe the effectiveness of our CNN at identifying EFL in two populations in whom the prevalence of EFL is different. This is an important experiment, because the prevalence of a condition in a population has a marked influence on the predictive power of a screening tool^31^. Based on accuracy alone, the CNN was equally effective at identifying EFL in GR_NORM_ and in GR_LOW_ (Table 2). However, in GR_NORM_, the CNN performed better at correctly predicting eFVLs that were non-flow-limited whereas it performed less well at predicting eFVLs with EFL (i.e. NPV higher than PPV). In contrast, in GR_LOW_, the CNN was slightly better at predicting eFVLs with EFL than eFVLs that were non-flow-limited (PPV higher than NPV). Akobeng conducted a quantitative analysis of the effect of disease prevalence on the PPV and NPV of an assessment test^31^. Whereas PPV progressively increases as the prevalence of a condition increases, NPV decreases as prevalence increases. Our findings for PPV and NPV in GR_NORM_ and GR_LOW_ are in-line with these effects of prevalence on predictive power. Our CNN was thus more effective at ruling-out EFL in persons with normal airway caliber and in whom there is a relatively low pre-test likelihood of EFL. In contrast, the CNN was better able to rule-in EFL in persons with narrowed airways and a higher pre-test likelihood of EFL.

The potential advantage of our deep learning approach for identifying EFL is that it does not require comparison of the eFVL with the MEFV curve, which has the tendency to overestimate EFL for several reasons. Firstly, excessive expiratory pressures during a forced expiration can compress thoracic gas such that the true limits for maximal airflow are underestimated and EFL will thus be overestimated^13^. Secondly, exercise causes bronchodilation in both healthy persons and in patients with asthma^2,3,14,32^. The unmeasured bronchodilation means that actual maximum expiratory flow during exercise might be more than measured by the baseline MEFV curve. Finally, accurate quantification of EFL requires precise measurement of operational lung volumes. The standard procedure whereby the eFVL is placed based on IC volume requires cooperation, and operational lung volumes will be overestimated if total lung capacity is not reached. The ability to automate identification of EFL based on the shape of the eFVL would improve efficiency, reduce errors, and be advantageous in clinical practice. We note that the negative expiratory pressure technique also does not require placement of an eFVL within a MEFV curve^33^. Yet, the method is technically demanding and difficult to implement, and has not seen widespread adoption by the scientific or clinical communities.

We used the pre-exercise MEFV curve to determine EFL in our subjects. Because of exercise bronchodilation, the extent of EFL is likely overestimated in our subjects^2^. The bronchodilation is plainly evident in Fig. 2C, where exercise expiratory flow exceeded the MEFV curve in GR_LOW_. The underestimated exercise maximum airflow would presumably increase the rate of false negatives (FN). That is, some of the eFVLs that were labelled as EFL were not, in reality, flow-limited during exercise when maximum expiratory flow was higher than measured at rest. Surprisingly, only 6.3% of the eFVLs labelled EFL in GR_LOW_ were misclassified as non-flow-limited (FN/[FN + TP] = 15/[15 + 212]) = 0.063). That only a small proportion of eFVLs were classified as FN might be related to the phenotype of the eFVL changing before it actually overlaps with the MEFV curve. It can be argued that dynamic airway narrowing begins before expiratory flow is truly limited^34^. Thus, the contour of the expiratory portion of the eFVL might begin assuming the flow-limited shape in breaths where expired flow approaches – but does not necessarily reach – the MEFV curve. Finally, in cases where tidal exercise airflow substantially exceeds the baseline MEFV curve, EFL might be achieved despite exercise bronchodilation.

### Clinical applicability

In clinical exercise testing, ventilatory limitation has historically been determined by comparing a patient's exercise ventilation with their maximum voluntary ventilation (i.e. breathing reserve). Reliance on this outcome is related to its ease of measurement and straight-forward interpretation. However, additional measures of ventilatory limitation provide important information beyond the somewhat crude measure of breathing reserve. More recently, analysis of operational lung volumes, breathing pattern, and the ventilatory equivalent for CO_2_ production have been integrated into identification of exercise ventilatory limitation^1^. While EFL clearly provides useful information regarding exertional dyspnea and exercise intolerance^1,34^, its analysis is not a routine component of clinical exercise testing. We think that the difficulty and nuance of measuring EFL is the primary reason that it is not a routine outcome in clinical exercise testing. While frank EFL will provide a mechanical limit to expiration, approaching EFL has been shown to alter breathing mechanics and ventilatory pattern, which then may contribute to dyspnea and exercise limitation^34^. Ultimately, an improved technological ability to identify and evaluate EFL in large numbers of patients will improve understanding of this physiology and thus how to apply it in different clinical contexts. For example, while EFL is historically best described as it relates to obstructive lung disease (specifically COPD), EFL is seen in a subset of patients with interstitial lung disease (ILD) and is also more prevalent in older adults^35,36^. The clinical implications of EFL in relation to ILD and aging remain largely undescribed but highlights the ways in which utilizing this information may be useful in clinical evaluation beyond obstructive lung disease.

### CNN model

Regarding the CNN model, the fact that we used a relatively simple CNN to identify EFL and it performed well without substantial fine tuning suggests that flow-volume curves are well-suited for classification by CNNs. Furthermore, by including a dropout layer and reducing the learning rate on plateaus in the loss metric, our model had safeguards in place to avoid overfitting. As is common with many deep learning approaches, the CNN model achieved good accuracy, but it does not provide “explainability.” In other words, while the model performed well at identifying EFL, it is not clear what it actually learned to perform its classifications. Additional investigations into the learned weights of the model could offer insights into the specific features of eFVLs the CNN learned as signals for the presence of EFL.

The size of the data set in this report (2931 eFVLs from 22 subjects) is smaller than normally recommended in neural network research. However, by design, our data set included eFVLs from healthy adults with normal spirometry and a slightly smaller number of subjects with mildly reduced spirometry. The data thus represent more than one population, and yet the model performed strongly in classifying EFL in all subjects. Importantly, a recent study found that increasing the size of a training data set does not lead to meaningful improvements in algorithm performance when the data are sufficiently representative of the target population^37^. The fact that our data includes eFVLs from persons exhibiting a range of baseline airway function leads us to expect that the model would perform well at classifying EFL in new subjects with similar ranges of baseline spirometry. Finally, it is important to acknowledge that application of deep machine learning in exercise and respiratory research is a very recent endeavor. There is much work to do to determine the potential of this technology in the analysis of exercise ventilatory constraints.

## Conclusions

We leveraged the power of modern machine learning by developing a CNN designed to identify EFL during exercise in a group of adults in whom airway function ranged from normal to mildly obstructed. Our CNN performed very well, demonstrating an overall accuracy of 90.6% at correctly identifying eFVLs that were flow-limited or not. This approach to identify EFL is predicated on analysis of the geometry formed by the relationship between expired volume and airflow during spontaneous breathing, streamlining the nuanced and cumbersome approach to identify EFL that requires placement of eFVLs within the MEFV curve. Ultimately, this approach could result in an objective method for identifying EFL and other exercise ventilatory constraints.

## Data Availability

The datasets generated during and/or analyzed during the current study are available from the corresponding author on reasonable request.
